# Prevalence of SARS-CoV-2 antibodies in Denmark: nationwide, population-based seroepidemiological study

**DOI:** 10.1007/s10654-021-00796-8

**Published:** 2021-08-22

**Authors:** Laura Espenhain, Siri Tribler, Charlotte Sværke Jørgensen, Christian Holm Hansen, Ute Wolff Sönksen, Steen Ethelberg

**Affiliations:** 1 Department of Infectious Disease Epidemiology and Prevention, Statens Serum Institut, Artillerivej 5, 2300 Copenhagen S, Denmark; 2 Department of Virus and Microbiological Special Diagnostics, Statens Serum Institut, Artillerivej 5, 2300 Copenhagen S, Denmark; 3 Department of Bacteria, Parasites and Fungi, Statens Serum Institut, Artillerivej 5, 2300 Copenhagen S, Denmark; 4 Department of Public Health, Global Health Section, University of Copenhagen, Øster Farimagsgade 5, 1014 Copenhagen K, Denmark

**Keywords:** Seroepidemiological studies, Population register, Questionnaire, COVID-19 serological testing, SARS-CoV-2

## Abstract

Seroprevalence studies have proven an important tool to monitor the progression of the coronavirus disease 2019 (COVID-19) pandemic. We present results of consecutive population-based seroprevalence surveys performed in Denmark in 2020. In spring, late summer and autumn/winter of 2020, invitation letters including a questionnaire covering symptoms were sent to representative samples of the population above 12 years and to parents of children below 18 years in the sample. Blood samples were analysed for total Ig and seroprevalence estimates per population segment were calculated and compared to other surveillance parameters. Based on 34 081 participants (participation rate 33%), seroprevalence estimates increased from 1.2% (95%CI: 0.3–1.9%) in May to 4.1% (95%CI: 3.1–4.9%) in December 2020. Seroprevalence estimates were roughly three times higher in those aged 12–29 years compared to 65 + and higher in metropolitan municipalities. By December 2020, 1.5% of the population had tested positive by RT-PCR. Infected individuals in older age groups were hospitalised several fold more often than in younger. Amongst seropositives, loss of taste/smell were the more specific symptoms, 32–56% did not report any symptoms. In more than half of seroconverted families, we did not see evidence of transmission between generations. Seroprevalence increased during 2020; adolescents were primarily infected in the autumn/winter. Denmark has a high per capita test rate; roughly one undiagnosed infection of SARS-CoV-2 were estimated to occur for each diagnosed case. Approximately half were asymptomatically infected. The epidemic appears to have progressed relatively modestly during 2020 in Denmark.

## Background

Severe acute respiratory syndrome coronavirus 2 (SARS-CoV-2) which causes COVID-19, manifests clinically ranging from asymptomatic infection to severe disease, which may lead to death. Surveillance of laboratory confirmed SARS-CoV-2 infections will capture only infected persons who are tested; which may only be a fraction of all cases in the community. The degree to which the COVID-19 pandemic is spreading through different countries or regions may therefore instead be assessed through population-based seroprevalence studies, which aim to quantify the proportion of the population that has developed antibodies against SARS-CoV-2. Such studies have been performed in a number of countries [1–9]. Some of these have been limited to specific geographic areas or been carried out on a non-representative sample of the population, but those designed as national representative surveys have found substantial geographical variability with higher seroprevalence in more densely populated areas [2, 7, 9, 10].

Similar to several other European countries, Denmark experienced increased transmission of SARS-CoV-2 infection in spring and late autumn 2020. A comprehensive lock-down was imposed in March 2020, gradually lifted towards summer and again gradually reintroduced during autumn and winter [11]. The Danish National Seroprevalence Survey of SARS-CoV-2 infection (DSS) was initiated in the spring of 2020, following a parliamentary decision in April 2020, which called for a representative population study to be performed. The study design was set by recommendation from two groups of independently appointed national experts in April 2020 [12, 13] and June 2020 [14] and the seroprevalence was subsequently determined at several time points throughout 2020. The aim of DSS was to follow the development of the COVID-19 epidemic by estimating the proportion of the population who had been infected with SARS-CoV-2, by age group, geography and sex at different time points, in order to guide preventive and control measures. Here we describe the set-up and results from the DSS in 2020 and relate the results to the national surveillance of RT-PCR diagnosed SARS-CoV-2-cases in Denmark.

## Methods

### Design and study population

DSS is a nationwide population-based prevalence survey aiming to investigate seroprevalence for SARS-CoV-2. The study should inform the national epidemic control in Denmark. It was launched in the spring of 2020 and performed by Statens Serum Institut (SSI) over three rounds: In May 2020 (DSS-I), August 2020 (DSS-II) and September-December 2020 (DSS-III).

In Denmark, a unique, personal civil registration number is assigned to all citizens, people with a residence permit, and people living outside Denmark but who are taxable in Denmark. For each survey round, a random population sample of Danish residents was drawn from the Danish civil registry [15] using the civil registration number. From the Danish civil registry, we also got information about age, sex and place of residence. For DSS-I, adults aged 18 years and older with an address in one of 30 municipalities which had a test facility (see below) at that time (n = 5), or were neighbouring a municipality with a test facility (n = 25) were eligible (approximately 45% of the population of Denmark). For DSS-II and DSS-III, people aged 12 years or older were selected by random sampling, with no restriction on municipality (n = 98). To make it a safe experience for children and motivate participation, parents living on the same address as invited children 12–17 years old, were also invited to have an antibody test. Parents’ antibody test results were not included in the seroprevalence calculation.

### Recruitment

Invitation letters (as pdf’s) were primarily sent via the secure, digital mailbox-system (“e-Boks”, the Danish digitalised postal system covering 90% of the Danish population [16]), using the civil registration number. For invitees exempt from receiving public mails in e-Boks (primarily elderly citizens) and invitees below 18 years of age, a physical letter was sent by regular mail. For DSS-I we invited a total of 5200 people, 2600 on May 5 and 2600 on May 15. For DSS-II we invited 6000 people each week on August 15, 21 and 28. For DSS-III we invited a total of 70 000 people over a 14 week period from September 11 to December 11, 2020. Letters of invitation contained information about: the aim and study design, the antibody test (how to interpret and understand the test result), and how to book a test. DSS-II and DSS-III also included a link to an electronic questionnaire. Invitations and questionnaires were available in Danish, English and Arabic language versions. The questionnaire contained, amongst others, questions about current and past symptoms.

### TestCenter Denmark, sample collection and analysis

Blood sampling was performed at test stations of ‘TestCenter Denmark’, a public national SARS-CoV-2 test facility system established during March and April 2020 [17, 18]. Nation-wide facilities offer free of charge, easy access RT-PCR testing for asymptomatic or mildly symptomatic individuals, in addition to the existing laboratories at the acute care hospitals that test symptomatic individuals and hospitalised patients upon admission. Booking of timeslots for RT-PCR testing was accessible for the population through a secure website and easily done on a smartphone. By May 2020, 22 RT-PCR-test facilities had been established, of which five facilities, one in each Danish region, were additionally equipped for taking blood samples. DSS-I made use of these five test stations. By August 2020, antibody testing could be carried out in 17 of the then 22 RT-PCR-test facilities, distributed throughout the whole country and were used for DSS-II and DSS-III. Study participants could book timeslots for antibody testing using the same IT-platform as for RT-PCR testing. Transportation to a test facility was at participants’ own cost. Blood sampling was performed by medically trained personnel. Five ml of blood were taken in BD Vacutainer® Serum tubes. The blood sample was packed, collected and transported to SSI for analysis. Samples were stored at 5°C until analysis. Total serum concentration of anti-SARS-CoV-2 immunoglobulin (total Ig) was measured by use of the Wantai SARS-CoV-2 Ab ELISA (Beijing Wantai Biological Pharmacy Enterprise, Beijing, China) according to the manufacturer’s instructions. The test had a sensitivity of 0.967 (CI95% 0.924–0.986) and a specificity of 0.995 (CI95% 0.987–0.998) [19].

### Data source and the Danish COVID-19 surveillance data

The Danish Microbiological Database (MiBa) contains all microbiological test results from all clinical microbiological departments in Denmark and microbiological and serological results from TestCenter Denmark [20]. Tests are registered in MiBa in a person identifiable format, by use of the civil registration number. Using MiBa we identified antibody test results and previous SARS-CoV-2 RT-PCR-test results amongst the study participants. From the Danish surveillance system of COVID-19 [21, 22] which is built on MiBa, we used information on the number of admitted and deceased RT-PCR-confirmed SARS-CoV-2 cases by date, sex and age group as well as the number of RT-PCR SARS-CoV-2 tests carried out. The Danish surveillance system of COVID-19 involves daily registry linkage to the National Patient Registry [23] for information about hospital admissions, and to the Civil Registry for information about sex and age, and The National Cause of Death Register [24] for information about deaths within 30 days for RT-PCR-diagnosed COVID-19 cases.

### Statistical analyses

We included all persons with a conclusive antibody test result within ten weeks from the invitation. We estimated the seroprevalence as the proportion of participating individuals with a positive antibody test result. We adjusted the seroprevalence estimates for test sensitivity and specificity using the Rogan-Gladen estimator [25] and computed 95% confidence intervals using Lang and Reiczigel’s method [26] which includes the accuracy of the sensitivity and specificity estimation. We present seroprevalence by age group, sex, the five geographical regions of Denmark and classification of municipality (capital, metropolitan, provincial, commuter, rural) as defined by Statistics Denmark [27] using information about the size of the biggest city and accessibility of workplaces. To evaluate whether the variation in participation rate by age groups, sex and region affected the estimated seroprevalence, missing sero-prevalence results were imputed based on multiple imputation using a logistic imputation model with sex, age group and region in the linear predictor in order to predict the missing 0 = negative/1 = positive response. The model was used to create 100 imputed datasets, i.e. 100 predictions were done for each of the missing test results, and the resulting prevalence estimates were then derived by combining estimates from these 100 datasets using Rubin’s rules [28].

The period of data collection for DSS-III was longer than that of DSS-I and DSS-II, spanning 14 weeks of which that later period coincided with the start of a second surge of infections. Therefore, we used two time point for reporting of DSS-III, based on when the blood samples were taken. We thus defined four time points (May, August, October and December), based on the date of blood sampling for the estimation of the seroprevalence. In order to match the narrower geographic and age inclusion criteria for DSS-I, we also estimated the seroprevalence restricted to the same criteria. We compared the estimates of infected individuals based on the seroprevalence to the number of RT-PCR test positive, hospitalised and deceased SARS-CoV-2 cases captured in national surveillance. To do that, we subtracted 14 days from the median date of blood sampling in each of the four periods to compare with RT-PCR-test positive from the national surveillance system. We added 10 days to find the comparable date for hospital admission and 20 days for number of deaths.

### Ethical and legal considerations

The DSS was performed as a national disease surveillance project, registered with the Danish Data Protection Agency and approved regarding legal, ethical and cyber-security issues by the SSI Compliance department in conjunction with the Danish governmental law firm. Participation was voluntary and invitees received information about the selection procedure, risks associated with participation, data security issues, their legal rights, including the right to withdraw from the study, and the use of their data and results in the letter of invitation.

## Results

### Participation

**Fig. 1:**
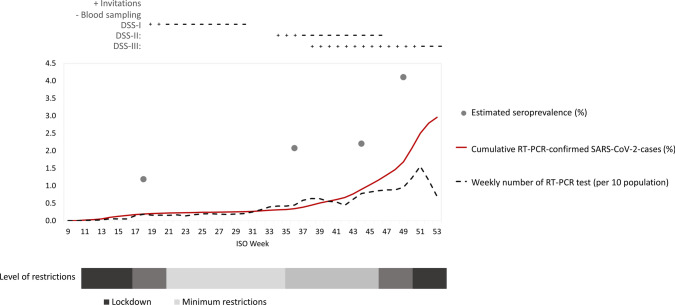
Seroprevalence point estimates per survey period (%, grey dots), cumulative RT-PCR-confirmed SARS-CoV-2 cases (%, solid line), weekly number of SARS-CoV-2 RT-PCR tests (per 10 population, dashed line), week of invitation for each survey (+ signs above the panel) and blood sampling (- signs above the panel) by ISO week, Denmark 2020. In addition, the timeline under the figure in schematic form illustrates the strength of the national measures that were in place to reduce transmission in 2020. In short, they comprised a full lockdown (shown using dark shading) involving a close-down of normal societal activity, but without imposing a curfew in March 2020 (week 11). The lockdown was gradually lifted from late April (week 16) into May. Over the summer, only comparatively mild restrictions were in place (regulating travel, gatherings, nightlife and more, shown using light shades). Starting September (week 34) restrictions were reintroduced and with an increasing incidence of infections occurring towards the end of the year. Harder restrictions were introduced in November (week 46) followed by a full lockdown being imposed in December (from week 50)

The three DSS study rounds had 2512 (48%), 7015 (39%), and 18 161 (26%) participants, respectively. Overall participation was lower in males and younger age groups (Table 1). For DSS-II and DSS-III respectively, the questionnaire was filled in by 2737 (39%) and 10 358 (57%) of the participants (Table 1). Eighty percent of those who participated did so within 12 days in DSS-I, within 31 days in DSS-II and within 20 days in DSS-III. The median dates of sampling for the four defined study periods were May 18 (referred to as ‘May’ below), September 19 (referred to as ‘August’), November 6 (‘October’), and December 16 (‘December’). Figure 1 shows the seroprevalence point estimates together with the invitation and data collection periods. For context, the figure further illustrates the cumulative COVID-19 incidence, weekly test intensity and level of national restrictions in Denmark in 2020. From Fig. 1 the smaller first wave and the bigger second wave can be seen and the rise in test intensity during 2020, where it peaked in week 51 (December) with more than 15 tests per 100 population per week, is apparent.

**Table 1:** Number of invited persons and proportion who participated by DSS, age group, sex, and region

Group		DSS-I^a^		DSS-II			DSS-III		
		Invited	Partici-pation (%)	Invited	Partici- pation (%)	% of participants who filled out questionnaire	Invited	Partici-pation (%)	% of participants who filled out questionnaire
Total		5200	(48)	18 000	(39)	(38)	70 000	(26)	(56)
*Age group*									
12–17 years				1492	(31)	(19)	5631	(20)	(41)
18–39 years		2146	(40)	5715	(32)	(31)	22 105	(22)	(47)
40–64 years		2077	(56)	6700	(48)	(42)	26 173	(33)	(61)
65 + years		977	(50)	4093	(36)	(46)	16 091	(22)	(64)
*Sex*									
Female		2585	(53)	9132	(44)	(40)	35 282	(29)	(59)
Male		2615	(44)	8868	(34)	(36)	34 718	(23)	(53)
*Region*									
Capital		2167	(48)	5680	(42)	(38)	22 268	(30)	(55)
Zealand		619	(43)	2618	(39)	(35)	10 107	(26)	(54)
Southern Denmark		798	(46)	3737	(35)	(39)	14 646	(20)	(60)
Mid Jutland		1035	(52)	4108	(38)	(42)	15 865	(23)	(61)
North Jutland		581	(52)	1857	(40)	(36)	7113	(29)	(53)
*Type of area (municipality)*									
Capital		2167	(48)	4985	(42)	(38)	19 436	(30)	(56)
Metropolitan		1526	(50)	2429	(43)	(37)	9260	(28)	(58)
Provincial		326	(44)	4070	(40)	(39)	15 570	(26)	(56)
Commuter		943	(48)	2848	(37)	(42)	11 393	(24)	(57)
Rural		238	(49)	3668	(33)	(37)	14 341	(21)	(56)

^a^Includes only people 18 years and older living in one of 30 municipalities which had a test facility at the time (n = 5) or was neighbouring a municipality with a test facility (n = 25). Approximately 45% of the population of Denmark

*DSS* Danish National Seroprevalence Survey of SARS-CoV-2 infection

### Seroprevalence

**Fig. 2:**
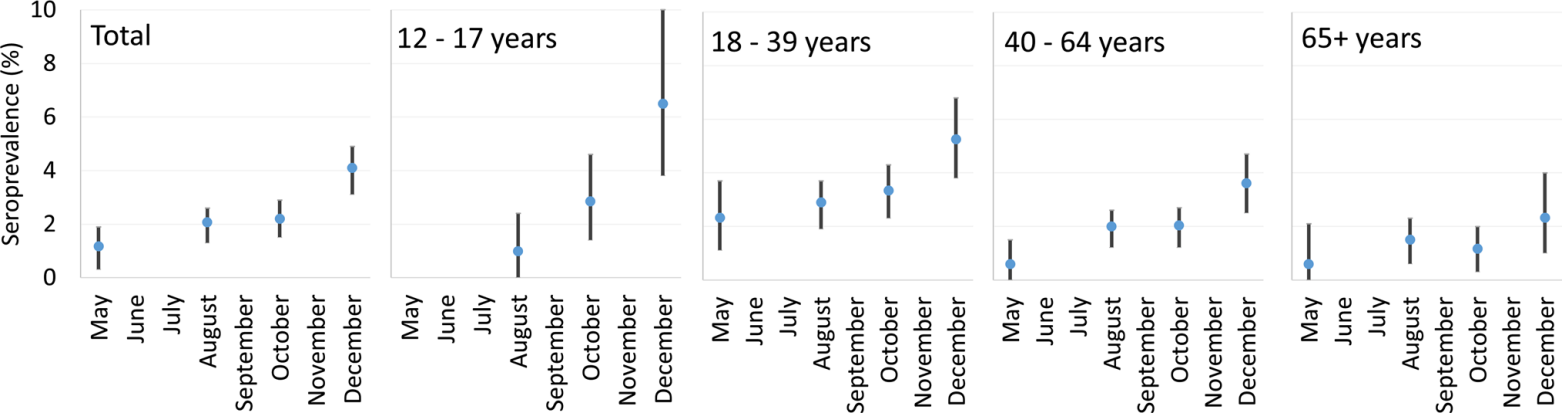
Seroprevalence and 95% confidence intervals of SARS-CoV-2 in May, August, October, and December 2020, by four age groups, Denmark

The proportion of participants with detectable SARS-CoV-2 antibodies increased from 1.2% (95%CI: 0.3%–1.9%) in May 2020 to 4.1% (95%CI: 3.1%–4.9%) in December 2020 (Table 2). Restricting the analysis to match the narrower geographic and age inclusion criteria for DSS-I, the estimated seroprevalence in December was 6.7% (95%CI 5.1%–8.4%). When taking the non-participation by age group, sex and region of residence into account by imputation, the estimates increased with up to 0.3 percentage points (Table 2). Point estimates tended to be higher in the two younger age groups (12–17 years and 18–39 years of age), lower in the 65 years and older age group (Fig. 2), and higher in the Capital region than in the other four regions. No difference was observed by sex (Table 2).

**Table 2:** Seroprevalence of SARS-CoV-2 in four periods (May, August, October, and December) 2020, by age group, sex, region and type of municipality

	May^a^ (n = 2512)		August (n = 11 478)		October (n = 9654)		December (n = 4044)	
	%	(95% CI)	%	(95% CI)	%	(95% CI)	%	(95% CI)
Total	1.2	(0.3–1.9)	2.1	(1.3–2.6)	2.2	(1.5–2.9)	4.1	(3.1–4.9)
Adjusted^b^ total	1.5	(0.9–2.1)	2.0	(1.7–2.4)	2.4	(1.9–2.8)	4.3	(3.4–5.1)
*Age group*								
12–17 years			1.0	(0–2.4)	2.9	(1.4–4.6)	6.5	(3.8–10)
18–39 years	2.3	(1.1–3.7)	2.9	(1.9–3.7)	3.3	(2.3–4.3)	5.3	(3.8–6.8)
40–64 years	0.6	(0.0–1.5)	2.0	(1.2–2.6)	2.0	(1.2–2.7)	3.6	(2.5–4.7)
65 + years	0.6	(0.0–2.1)	1.5	(0.6–2.3)	1.2	(0.3–2.0)	2.3	(1.0–4.0)
*Sex*								
Female	0.9	(0.3–1.8)	2.1	(1.3–2.8)	2.0	(1.2–2.7)	4.2	(3.0–5.2)
Male	1.5	(0.5–2.6)	2.0	(1.1–2.6)	2.6	(1.7–3.3)	4.0	(2.8–5.2)
*Region*								
Capital	1.8	(0.7–3.0)	3.2	(2.3–4.0)	3.3	(2.3–4.1)	5.0	(3.7–6.3)
Zealand	1.9	(0.2–4.6)	1.9	(0.9–2.8)	1.4	(0.4–2.3)	4.0	(2.3–6.1)
South	0.7	(0–2.5)	1.6	(0.6–2.4)	1.7	(0.7–2.6)	3.3	(1.8–5.0)
Mid Jutland	0.3	(0–1.6)	1.3	(0.4–2.0)	2.0	(1.0–2.9)	3.6	(2.1- 5.3)
North Jutland	0.6	(0–2.6)	1.2	(0.3–2.2)	1.6	(0.5–2.7)	3.1	(1.3–5.5)
*Type of area (municipality)*								
Capital	1.8	(0.7–2.9)	3.3	(2.3–4.1)	3.5	(2.5–4.4)	5.6	(4.2–7.1)
Metropolitan	0.9	(0–2.1)	1.8	(0.8–2.7)	1.5	(0.5–2.5)	4.9	(3.1–7.1)
Provincial	3.2	(0.7–8.0)	1.5	(0.6–2.3)	2.3	(1.3–3.2)	3.6	(2.1–5.1)
Commuter	0	(0–1.3)	1.9	(0.9–2.8)	0.8	(0–1.6)	3.3	(1.8–5.3)
Rural	0	(0–1.2)	1.0	(0.1–1.8)	1.8	(0.8–2.7)	1.3	(0.2–2.9)

^a^Includes only people 18 years and older living in one of 30 municipalities which had a test facility at the time (n = 5) or was neighbouring a municipality with a test facility (n = 25). Approximately 45% of the population of Denmark

^**b**^Adjusted for non-participation by sex, age group and region by multiple imputation

### Seroprevalence of children and their parents

A total of 1244 families had a child and at least one parent tested. Among these, 79 (6.4%) families were found to have at least one seropositive family member. These included 3.2% with a seropositive child and 4.2% families with at least one seropositive parent. In 21 of the 79 seropositive families (27%), both child and parent(s) were seropositive. In 19 families (24%) only the child was seropositive, and in 39 (49%) seropositive families the child was seronegative.

### Symptoms amongst seropositive participants

Of the 369 seropositive participants who filled in the questionnaire, 59% reported having experienced at least one of four core symptoms (fever, cough, shortness of breath and/or loss of sense of taste or smell) since February 2020, versus 28% amongst seronegative participants (Table 3). Loss of smell or taste, reported by 28% of the seropositives, was highly associated with seropositivity (prevalence ratio of 9.8, Table 3).

**Table 3:** Number, proportion (%) of participants and prevalence ratio of symptoms, by antibody status, DSS-II and DSS-III, Denmark 2020

Symptom^a^	Antibody test result					
	Positive (n = 369)		Negative (n = 12 726)		Prevalence ratio	95%CI
	Number	Proportion (%)	Number	Proportion (%)		
No symptoms reported	112	(30)	7232	(57)	0.53	(0.46–0.62)
At least one core symptom^b^	216	(59)	3508	(28)	2.1	(1.9–2.3)
Fever	139	(38)	1801	(14)	2.7	(2.3–3.1)
Cough	141	(38)	2544	(20)	1.9	(1.7–2.2)
Shortness of breath	71	(20)	811	(6)	2.8	(2.2–3.5)
Loss of sense of smell or taste	104	(28)	367	(3)	9.8	(8.1–12)
Other symptoms^c^	41	(11)	1986	(16)	0.71	(0.53–0.95)

^a^In the period since 1 February 2020

^b^Core symptoms includes one or more of the following: fever, cough, shortness of breath and/or loss of sense of taste or smell

^c^Muscle ache, eye pain, head ache, coloured sputum, runny nose, sneezing, back pain, tiredness *without* one of the core symptoms

DSS: Danish National Seroprevalence Survey of SARS-CoV-2 infection

In DSS-II 44% (95%CI: 32–56%) of the seropositive participants reported no symptoms since February and a further 9% reported symptoms not belonging to the five mentioned core symptoms. In DSS-III the equivalent figures were 27% and 11%.

### Previous RT-PCR positive participants

A total of 255 participants had had a positive RT-PCR-test result prior to the antibody test. Of those, 232 had an antibody test 14 days or more after the positive RT-PCR result and SARS-CoV-2 antibodies were detected in 217 (97% when adjusting for the sensitivity and specificity of the test) whereas 15 did not have detectable antibodies. The median time in days between the positive RT-PCR result and the positive antibody test result, amongst those with at least 14 days between the two, was 56 days [range: 14–293] and 95 days [range: 17–188] for the 15 seronegative persons.

### Seroprevalence in relation to RT-PCR-diagnosed, admitted and deceased SARS-CoV-2 cases from national surveillance

According to the national COVID-19 surveillance system, 78 125 persons above 12 years (1.53/100 inhabitants) had tested positive for SARS-CoV-2 by RT-PCR in Denmark by December 2 (Fig. 1). Our finding of a seroprevalence of 4.1% (95%CI 3.1–4.9) corresponds to a total of 208 000 (157 000–249 000) persons above 12 years of age having been infected in Denmark by December 2, 2020. Thus the estimated ratio of infected persons to RT-PCR diagnosed persons captured in the national surveillance system was six in May 2020 and two in December (Table 4). The estimated ratio varied by age. It was higher in the 18–39 year age group in May and August and decreased during autumn. No obvious pattern was seen for the 65-year and older age group or sex during the period (Table 4). The infection fatality rate and rate of admitted as a proportion of the estimated number of infected increased markedly with older age and was 3.8% in the 65-year and older age group (Table 4).

**Table 4:** Ratio between estimated number of infected and RT-PCR-diagnosed SARS-CoV-2 cases from national surveillance at four time points and number of SARS-CoV-2 admissions, infection admission rate, number of SARS-CoV-2 deaths and infection fatality rate per December 2020, by age group and sex, Denmark 2020

	Estimated ratio of infected/PCR-diagnosed COVID-19 cases								
	May^a^	August	October	December	Estimated number of infected by December 2	Admissions by December 12^b^	IAR (%)	Deaths by December 22^b^	IFR (%)
Total	6	6	3	2	208 000	5987	2.9	1081	0.52
*Age group*									
12–17 years	–	5	4	3	26 000	58	0.22	0	–
18–39 years	13	7	4	2	84 000	642	0.76	3	0.00
40–64 years	3	5	3	2	69 000	2033	3.0	56	0.08
65 + years	3	6	3	3	27 000	3164	12	1022	3.8
*Sex*									
Female	4	6	3	2	107 000	2790	2.6	480	0.45
Male	9	6	4	2	101 000	3107	3.1	601	0.60

^a^Includes only people 18 years and older living in one of 30 municipalities which had a test facility at the time (n = 5) or was neighbouring a municipality with a test facility (n = 25). Approximately 45% of the population of Denmark

^b^Among RT-PCR confirmed SARS-CoV-2 cases from national surveillance

IAR = Infection admission rate. IFR = Infection fatality rate

## Discussion

In this national representative seroprevalence study amongst Danish residents aged 12 years and older, we found detectable SARS-CoV-2 antibodies in 4.1% (95%CI: 3.1–4.9%) of the participants by the beginning of December 2020. This was four times more than the estimate from May and twice the estimate of August 2020. This study also provides information on the regional and demographic progression of the epidemic and the results can be interpreted in the context of other surveillance parameters.

The seroprevalence varied by geography and age group, which is consistent with the general picture from the national surveillance system. It appears that people from more densely populated urban areas were infected in the early stage of the epidemic, and that the epidemic later gradually spread to the less densely populated areas. Our results are in line with the serological surveys of blood donors which have also been carried out in Denmark [29], although this group may not be representative of the general population.

Between May and December 2020, RT-PCR testing rose from 15 up to 150 tests per 1000 inhabitants per week. Our results show that the for each RT-PCR-confirmed SARS-CoV-2-case captured by the national surveillance system there were five undetected infections in the spring but just a little more than 1 by early December 2020. There were relatively more undiagnosed cases in the 18–39-year age group during the first six month of the epidemic, possibly because this age group experiences a less severe course of disease. It is well established that severity of illness increases markedly with age [30] and the estimated infection fatality and infection admission rates also increased markedly by age group in this study. Alternative use of a 60-day mortality measure instead of a 30-day measure did not change the infection fatality estimate markedly (0.52 vs. 0.58).

National representative seroprevalence surveys from other countries have shown variable seroprevalence estimates [31]: Surveys in France [10] in May, and Spain [2] and Brazil [9] in June and the Netherlands (7) in July 2020 estimated that respectively around 4.5%, 5%, 3.5% and 4% of the population had been infected with SARS-CoV-2 at these timepoints. All four surveys revealed substantial geographical variability. In September 2020, a survey carried out in the US found that in 25 of 49 jurisdictions with sufficient samples to estimate seroprevalence, more than 5% of people had detectable SARS-CoV-2 antibodies [1]. Our results are comparatively low and thus the epidemic appears to have affected Denmark only mildly in 2020. This may be corroborated by the cumulated mortality numbers, which by 31 Dec 2020 were 23 per 100 000 [21], a low number relative to most other European countries [32].

Estimates from other studies [33, 34] of the proportion of asymptomatic infections out of the total number of SARS-CoV-2 infections vary notably from a few percent to 41% with a pooled overall proportion of 17% found in a recent meta-analysis [35]. In DSS-II, carried out in the late summer, 44% of the seropositive participants, who filled out the questionnaire, did not recall having had any symptoms of acute infection since February 2020 and an additional 9% reported symptoms not typically associated with a SARS-CoV-2 infection. The percentage reporting no symptoms since February 2020 fell to 27% in the DSS-III. However at this point other respiratory illnesses may increasingly have affected the results. Thus, our best estimate is that around 41% of the seropositive persons experienced asymptomatic infections. The two younger age groups were less likely to have filled out the questionnaire. Because these groups are more likely to experience asymptomatic infections, the overall proportion of asymptomatic infections may be underestimated. We found that loss of smell or taste, experienced by 28% with SARS-CoV-2 antibodies, were by far the more specific symptoms of SARS-CoV-2 infection.

In more than two thirds of families with at least one seropositive family member, only the parent(s) or the child had seroconverted. Though household exposure is a strong risk factor for SARS-CoV-2 infection [36, 37], this finding indicates that transmission between generations within households is the exception rather than the rule. This is in line with a previous meta-analysis which have estimated a secondary attack rate of 16.6% (95% CI, 14.0–19.3%) [38] and with two register-based studies carried out in Denmark where secondary attack rates between 10%-30% were found [11, 39]. However, a Norwegian study found an overall attack rate of 45% in households by use of serology [40]. We were unable to disentangle the direction of transmission between generations and our design did not allow us to shed light on seroconversion of siblings or other household members who were not the legal parent of the child.

Though numbers were low, we found that < 5% of previous RT-PCR positive participants did not have detectable SARS-CoV-2-antibodies at least 14 days after their first positive RT-PCR test. This might be because of waning immunity, or that the individuals did not elicit a detectable antibody response (possibly due to mild or asymptomatic infection). The proportion does correspond to what has been reported from Iceland [41] and a study from the UK [6].

Denmark has a relatively high degree of IT penetration and frequently makes use of national registers and public digital resources. Utilisation hereof was amongst the strengths of this study. From the Danish national civil register, it was possible to obtain a random sample of residents, and identify the parents of those below 18 years of age. Individually referable national surveillance data allowed us to identify all previous RT-PCR SARS-CoV-2 tests amongst the participants and relate this to their antibody-status. Another strength of the study was the use of the logistical set-up of the large state-driven nation-wide test-system, TestCenter Denmark, that was created as a parallel system to the existing clinical test system located at the hospitals. Using the existing set up for RT-PCR testing also for taking blood samples, meant that most people had an easy access to a test facility for antibody testing.

The study had several limitations. When interpreting the findings, the suboptimal participation and response rates should be taken into account. Participation decreased from 48% in DSS-I, through 39% in DSS-II to 26% in DSS-III, and even fewer replied to the questionnaire concerning symptoms. It is possible that the low questionnaire response rate may be related to the lay-out of the invitation letter, which generally highlighted the blood test. Also to be remembered is that even though the drawn sample is representative of the population, participation may not be. The seroprevalence estimates were stable but slightly underestimated when taking the non-participation by age group, sex and region of residence into account. However, we do not know if certain subgroups of the population were underrepresented in the study, but expect that factors such as distance to the nearest test facility, existing illness or immobility at the time of the invitation and ethnic background could have affected participation. In Denmark, some population groups with immigrant background are known to have been overrepresented among SARS-CoV-2 cases [42]. If such groups had relatively lower participation rates in our study, there would be a tendency for an under-estimation of the true seroprevalence. Due to the geographical distribution of the test stations, some persons had quite long driving distances to a test facility (up to 100 km for some), which may have affected their willingness to participate in the study. If people living in remote areas far from a test station had relatively lower participation rates, we may reversely have overestimated the true seroprevalence. Waning of antibodies may also have led us to underestimate the true seroprevalence. A waning effect would have been more likely to affect participants infected early in the pandemic and possibly primarily those with few or mild symptoms. In our study, we found antibodies in 95% of the participants that had previously tested positive by RT-PCR.

In conclusion, our study provides estimates of SARS-CoV-2 dissemination in Denmark at four time points during 2020, based on a representative sample of the population and relate these to the number of RT-PCR-confirmed SARS-CoV-2 cases in the national surveillance system. We found that the epidemic had predominantly affected the capital and metropolitan areas and saw indications of a higher seroprevalence in young adults throughout the epidemic, although children 12–17 years old were mainly affected in the second surge of the epidemic. Overall, the estimated seroprevalence in Denmark throughout 2020 was low, compared to other countries. The results seem to support that the measures introduced in Denmark in the spring of 2020 and onwards have been effective in keeping the epidemic from developing rapidly in the community, however also indicate that the majority of the population was still at risk of contracting SARS-CoV-2 infection at the end of 2020. As almost half of the infections seem to be asymptomatic, social distance measures and efforts to identify and isolate new cases and their contacts are imperative for future epidemic control in an unvaccinated population.

## Data Availability

Data is not publicly available. Code is not publicly available.
